# Engineering BN‐Doped Open‐Shell Polycyclic Hydrocarbons for Enhanced Photothermal Conversion and Therapy

**DOI:** 10.1002/anie.9715423

**Published:** 2026-07-11

**Authors:** Peiyun Zeng, Ziqi Zhuang, Huan Luo, Yubin Fu, Peiyu Wang, Yating Zheng, Gang Liu, Junzhi Liu

**Affiliations:** ^1^ Department of Chemistry The University of Hong Kong Hong Kong China; ^2^ Chemistry and Chemical Engineering Guangdong Laboratory Shantou China; ^3^ State Key Laboratory of Vaccines for Infectious Diseases Center for Molecular Imaging and Translational Medicine Xiang An Biomedicine Laboratory National Innovation Platform for Industry‐Education Integration in Vaccine Research School of Public Health Xiamen University Xiamen China; ^4^ School of Energy and Environment Southeast University Nanjing China; ^5^ State Key Laboratory of Synthetic Chemistry HKU‐CAS Joint Laboratory on New Materials and Shanghai‐Hong Kong Joint Laboratory on Chemical Synthesis The University of Hong Kong Hong Kong China; ^6^ Materials Innovation Institute for Life Sciences and Energy (MILES) HKU‐SIRI Shenzhen China

**Keywords:** BN‐doped, open‐shell character, photothermal therapy, polycyclic hydrocarbons, tetraradicaloid

## Abstract

Traditional inorganic photothermal agents (PTAs) suffer from poor biodegradability, while undoped carbon‐based materials exhibit low photothermal conversion efficiency (PCE). In contrast to the established organic PTAs with donor–acceptor architectures, herein, we developed a new class of atomically precise BN‐doped polycyclic hydrocarbons (PHs) featuring stable diradicaloid (**BN‐Dira**: *y*_0_ = 0.74) and tetraradicaloid (**BN‐Tetra**: *y*_0_ = 0.43, *y*_1_ = 0.30) character for high‐efficiency photothermal therapy (PTT). Our design is based on the synergistic integration of 1,2‐BN units and radical engineering within an expanded π‐conjugated skeleton. The complementary electron‐donating (N) and electron‐accepting (B) sites effectively modulate the intramolecular charge distribution, which promotes effective electron delocalization. The resulting compounds exhibit exceptional ambient stability, with the tetraradicaloid species demonstrating half‐lives of over 1 year. In addition, the engineered compound **BN‐Dira** exhibit high NIR absorption and PCE of 83.6%. In vitro, they induce rapid cancer cell apoptosis under NIR irradiation (808 nm). In vivo murine models confirm efficient tumor eradication with minimal side effects. This work establishes a new protocol for stable, radical‐based PH PTAs, providing a solution for high‐performance cancer therapies that can be translated into clinical applications.

## Graphical abstract

Two novel bonded BN‐doped polycyclic hydrocarbons **BN‐Dira** and **BN‐Tetra** with open‐shell character were rationally designed and synthesized. Their molecular structures were unambiguously confirmed by crystallographic analysis. Facilitated by their intense absorption in the NIR region, high PCE (83.6%) and good photothermal stability, **BN‐Dira** and **BN‐Tetra** have been employed as photothermal agents, demonstrating highly efficient photothermal ablation in vitro and in vivo.

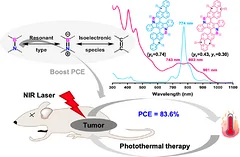

## 1 Introduction

Cancer persists as a defining global health challenge, with conventional chemotherapy and radiotherapy often burdened by severe off‐target toxicity due to their inherent lack of specificity [1, 2]. In response, photothermal therapy (PTT) has emerged as a highly promising minimally invasive alternative [3, 4, 5, 6]. PTT utilizes photothermal agents (PTAs) to absorb light energy, particularly within the biologically transparent near‐infrared (NIR) window (700–1000 nm), converting it into localized heat to selectively ablate malignant cells while preserving healthy tissues [7, 8, 9, 10, 11]. Despite its considerable potential, the clinical translation of PTT critically depends on developing PTAs that can simultaneously achieve high photothermal conversion efficiency (PCE) and adequate biocompatibility [12, 13, 14].

Traditional inorganic PTAs, such as gold nanoparticles [15, 16] and graphene oxide [17], frequently encounter limitations related to poor biodegradability and potential long‐term toxicity concerns. This has driven significant interest toward carbon‐based nanomaterials, specifically carbon dots (CDs). CDs offer compelling advantages, including inherent biocompatibility, facile surface functionalization, and tunable optical properties derived from their sp^2^/sp^3^ hybridized carbon cores and surface groups [18, 19, 20]. However, the application of conventional CDs in PTT fundamentally constrained by their typically modest PCE, stemming from inefficient non‐radiative electron–hole recombination pathways. Efforts to boost the PCE of CDs have largely focused on heteroatom doping (e.g., N, S, P, B) to perturb the electronic structures and accelerate non‐radiative decay [21]. While effective in principle, this approach presents substantial challenges. The type, concentration, and position of dopants critically influence the resulting properties, but precise synthetic control over these parameters remains elusive. Uncontrolled doping can introduce detrimental effects such as aggregation (excessive N), reduced photostability (P), or formation of oxidized byproducts. Furthermore, traditional metal doping (Fe, Cu, Ag), while enhancing electron transfer or adding magnetic properties, is largely precluded by toxicity concerns [22]. Consequently, achieving the necessary strong NIR absorbance and high PCE through conventional heteroatom doping alone has proven truly difficult, highlighting an urgent need for a novel design principle.

**FIGURE 1:**
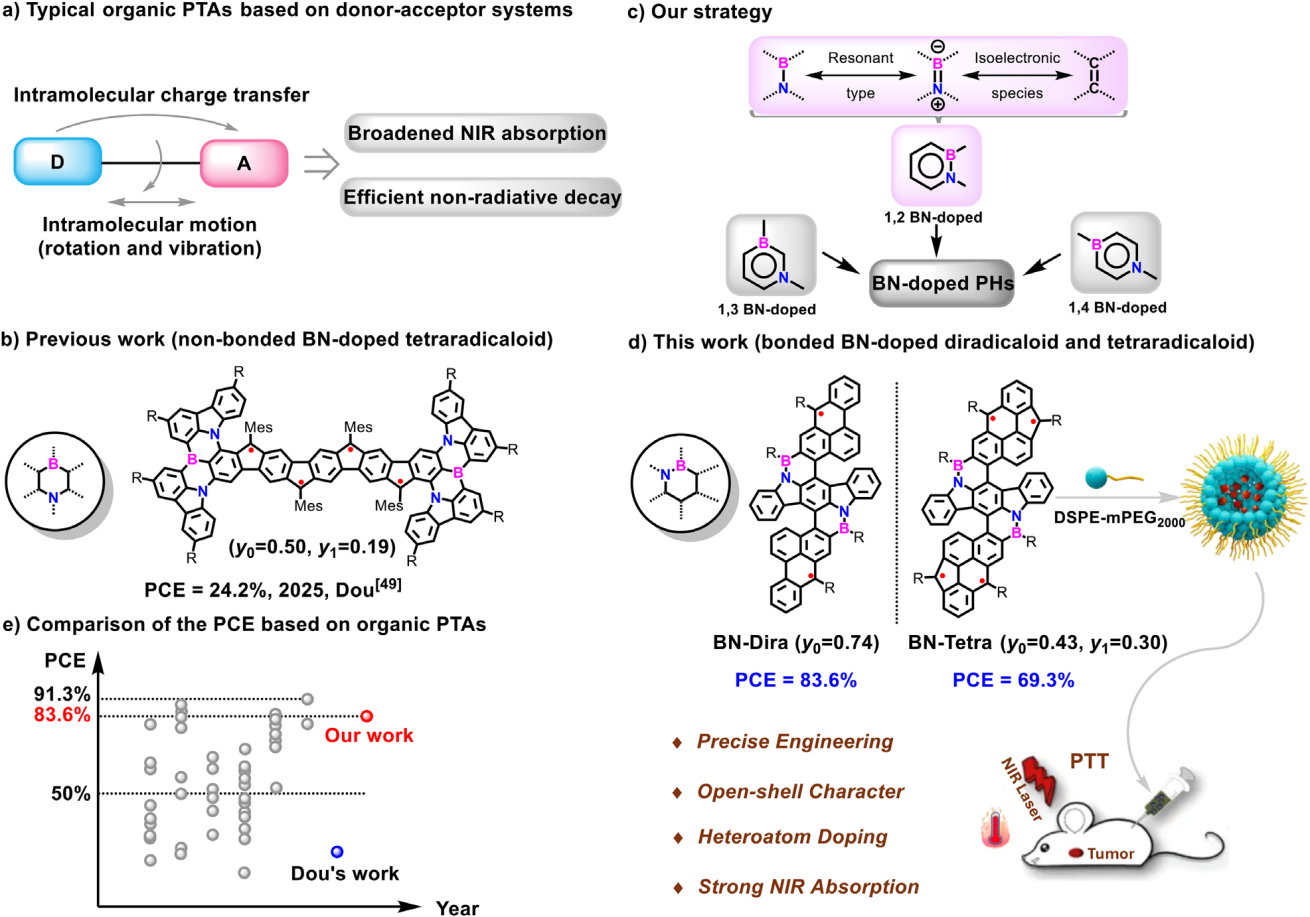
(a) Typical organic PTAs based on donor–acceptor systems. (b) Previously reported non‐bonded BN‐doped polycyclic hydrocarbon for PTT. (c) Our strategy. (d) Schematic illustration of water‐dispersed radicaloid nanoparticles (**BN‐Dira** and **BN‐Tetra**) with boosted PCE for enhanced PTT (This work). (e) Comprehensive comparison of the PCE based on organic PTAs in recent years.

A widely adopted strategy employs organic PTAs featuring donor–acceptor (D–A) architectures, which enable NIR absorption through intermolecular charge transfer and achieve high PCE via enhanced nonradiative relaxation pathways (Figure 1a) [3, 23, 24]. Several D–A systems have demonstrated exceptionally high PCEs exceeding 80%, underscoring their effectiveness as PTAs for PTT [25, 26, 27, 28, 29, 30]. Another promising alternative strategy involves harnessing radical chemistry to engineer open‐shell electronic structures within CDs [31, 32, 33, 34]. In open‐shell systems, the energy gap between the singly occupied molecular orbital (SOMO) and the singly unoccupied molecular orbital (SUMO) is typically lower than the HOMO–LUMO gap in closed‐shell structures [35, 36, 37, 38]. This narrowing of the effective energy gap can dramatically enhance NIR light absorption and promote non‐radiative decay pathways essential for efficient heat generation. Stable organic radicals (e.g., nitroxides, carbon‐centered radicals) can introduce mid‐gap states, further broaden absorption and increase the probability of heat‐dissipating electron transitions. Crucially, radical‐mediated engineering offers the potential to significantly boost PCE while preserving the inherent biocompatibility and scalability advantages of CDs. However, the practical realization of radical‐functionalized CDs as viable PTAs has been hampered by two major obstacles: (1) the inherent difficulty in achieving atomic‐level precision over size, structure, and radical positioning using traditional methods (leading to heterogeneity and suboptimal performance); and (2) the notorious instability of many radical species under physiological conditions [39, 40, 41, 42, 43, 44, 45]. Very recently, Dou and coworkers reported the design of a 1,4‐BN‐doped (non‐bonded) polycyclic hydrocarbon (PH) exhibiting open‐shell singlet ground state character, with a partial tetraradical contribution to the ground state (Figure 1b) [46, 47, 48]. However, the resulting PCE reached only 24.2 % [48]. Despite this advance, utilizing the open‐shell character to the fullest extent with an efficiently higher PCE remains a key challenge.

Leveraging the cooperative effect of boron and nitrogen atoms to modulate the electronic structure of the PHs [49], a new type of BN‐doped PHs, namely, bonded BN‐doped PHs, has emerged, enabling precise tuning the diradical character. Herein, we present an approach that overcomes above obstacles by integrating precise carbon‐based nanomaterial engineering with advanced radical chemistry. Utilizing the distinct physicochemical properties of 1,2‐BN units, which are isoelectronic to but electronically distinct from C═C double bonds, we report the atomically precise synthesis of novel bonded BN‑doped PHs featuring stable diradical (**BN‑Dira**: *y*_0_ = 0.74) and tetraradical (**BN‑Tetra**: *y*_0_ = 0.43, *y*_1_ = 0.30) character (Figure 1c,d). Our strategy involves two key elements: (1) substituting specific carbon atoms with electron‐donating N (lone pair) and electron‐accepting B (empty orbital) to strategically alter charge distribution and electronic properties [39, 50, 51, 52, 53, 54], and (2) deliberately modifying the conjugated backbone by shifting spin delocalization from six‐membered to combined five‐ and six‐membered rings while expanding the overall π‐conjugated skeleton. This enhanced conjugation promotes efficient electron delocalization and radical stability (with a half‐life exceeding 1 year for **BN‑Tetra**). Electron paramagnetic resonance (EPR) spectroscopy unambiguously confirms the open‐shell nature of these two engineered PHs. Both these diradicaloid and tetraradicaloid PHs exhibit strong NIR absorption and high PCE which the molar absorption coefficient (*ε*) of **BN‑Dira** was determined as high as 137 346 M^−1^cm^−1^ at 774 nm and its PCE impressively reached 83.6%. This PCE value is not only among the highest reported for organic NIR‑I PTT agents (Figure 1e), but also higher (3.5 times) than previously reported non‐bonded BN‐doped PH (Figure 1b) [48]. Evaluated as PTAs, these PHs demonstrate rapid and potent heat generation in vitro, inducing significant apoptosis in cancer cell lines under NIR irradiation (808 nm). Critically, in vivo murine models confirm highly efficient tumor eradication with minimal side effects, showcasing their therapeutic efficacy and biocompatibility. This work presents stable PH PTAs with radical character via bonded BN engineering, enabling high‐performance, biocompatible cancer therapy, and providing a versatile protocol for next‐generation, personalized nanomedicine.

## 2 Results and Discussion

**FIGURE 2:**
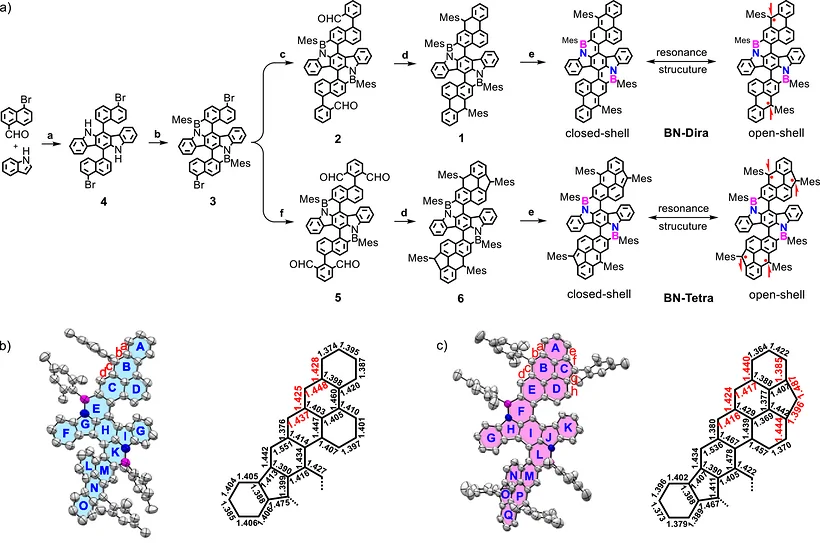
(a) Synthetic routes toward two polycyclic hydrocarbons **BN‐Dira** and **BN‐Tetra**. Reaction conditions: a: HI, toluene, 110^0^C, 12 h, 50%; b: BBr_3_, MesMgBr, toluene, 110°C, 12 h, 30%; c: (2‐Formylphenyl)boronic acid, Pd(PPh_3_)_4_, K_2_CO_3_, dioxane/H_2_O, 24 h, 50%; d: (1) MesMgBr, THF, (2) BF_3_•OEt_2_, DCM, 80%; e: *t*‐BuOK, THF, 80%; f: 2‐(4,4,5,5‐Tetramethyl‐1,3,2‐dioxaborolan‐2‐yl)isophthalaldehyde, Pd(OAc)_2_, SPhos, K_3_PO_4_, dioxane/H_2_O, 90^0^C, 12 h, 40%. (b) Single‐crystal structure and bond length of **BN‐Dira**. (c) Single‐crystal structure and bond length of **BN‐Tetra**.

Two radical‐functionalized PHs (**BN‐Dira** and **BN‐Tetra**) containing bonded B and N units were synthesized via rational designs and mature synthetic routes (Figure 2a). The first condensation reaction, followed by borylation, afforded compound **3**. The two‐fold Suzuki coupling of compound **3** with (2‐formylphenyl)boronic acid yielded compound **2**. Precursor **1** was synthesized through a sequential procedure involving nucleophilic addition between compound **2** and Grignard reagent, followed by an intramolecular Friedel‐Craft alkylation. The final oxidation of precursor **1** afforded PH **BN‐Dira** in 80% yield. Simultaneously, replacement of (2‐formylphenyl)boronic acid with 2‐(4,4,5,5‐tetramethyl‐1,3,2‐dioxaborolan‐2‐yl)isophthalaldehyde in the Suzuki coupling yielded compound **5**. Compound **5** was converted to compound **6** through a nucleophilic addition followed by a Friedel‐Craft alkylation. Subsequent oxidation of **6** then yielded PH **BN‐Tetra**. The chemical structures of these two PHs were unambiguously confirmed with NMR spectroscopy and high‐resolution mass spectrometry (Figures S1–S26).

**FIGURE 3:**
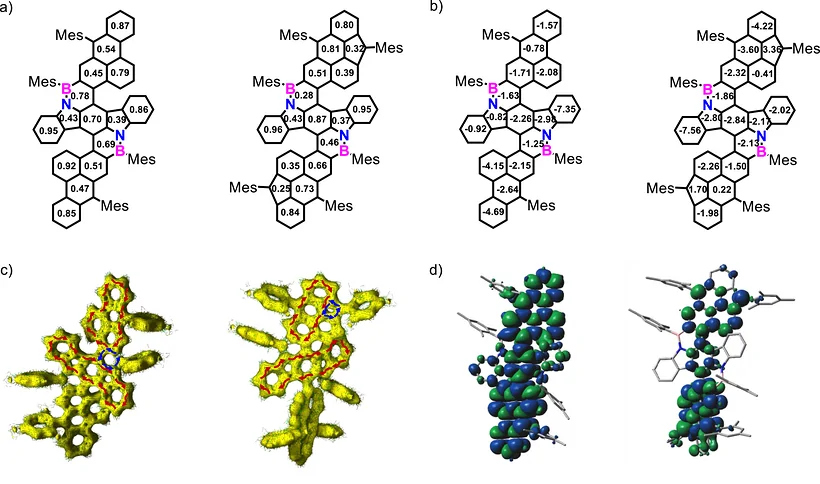
(a) Calculated HOMA values, (b) calculated NICS (0) values, (c) the diamagnetic (clockwise), and paramagnetic (counterclockwise) ring currents under a magnetic field parallel to the *z*‐axis are highlighted by red and blue arrows in AICD plots, and (d) calculated spin distribution map of **BN‐Dira** and **BN‐Tetra**.

The molecular structures of these two PHs were unambiguously confirmed by single‐crystal x‐ray analysis (Figure 2b,c) [55]. In their crystal packing, no π–π interaction was observed in both **BN‐Dira** and **BN‐Tetra** due to the steric hindrance from bulky substituents (Figures 2d,e, and S29 and S30). Single‐crystal analysis of **BN‐Dira** revealed negligible bond length alternation along the zigzag edge (bonds a–d, 1.425–1.448 Å, Figure 2b), suggesting effective electron (spin) delocalization (Figure 3d) and open‐shell contribution to the ground state. Similarly, the bond‐length along the zigzag edges in **BN‐Tetra** (bonds a–d (1.416–1.440 Å) and e–f (1.385–1.487 Å), Figure 2c) are longer than a typical double bond (≈1.33 Å), indicates the double‐bond characteristics of bonds are greatly weakened due to the open‐shell contribution to the ground state. Compared to **BN‐Dira**, **BN‐Tetra** demonstrated reduced electron (spin) delocalization (Figure 3d). To further evaluate the aromaticity, harmonic oscillator model of aromaticity (HOMA) analysis was performed for each ring. Notably, pronounced aromaticity was observed in rings A/D/F/H/J/M/O of **BN‐Dira** (Figure 3a) and rings A/B/G/I/K/P/Q of **BN‐Tetra** (Figure 3a), as indicated by their large HOMA values. In contrast, the rest of the rings in **BN‐Dira** and **BN‐Tetra** showed modest HOMA values. The localized aromaticity derived from these observations allowed for the proposing resonance structures of **BN‐Dira** and **BN‐Tetra** (Figure S33), which also align with their calculated spin distribution maps (Figure 3d). Additionally, nucleus‐independent chemical shift (NICS) calculations and anisotropy of the induced current density (AICD) of **BN‐Dira** and **BN‐Tetra** (Figure 3b,c) corroborate the aromatic trends observed in their HOMA analysis.

**FIGURE 4:**
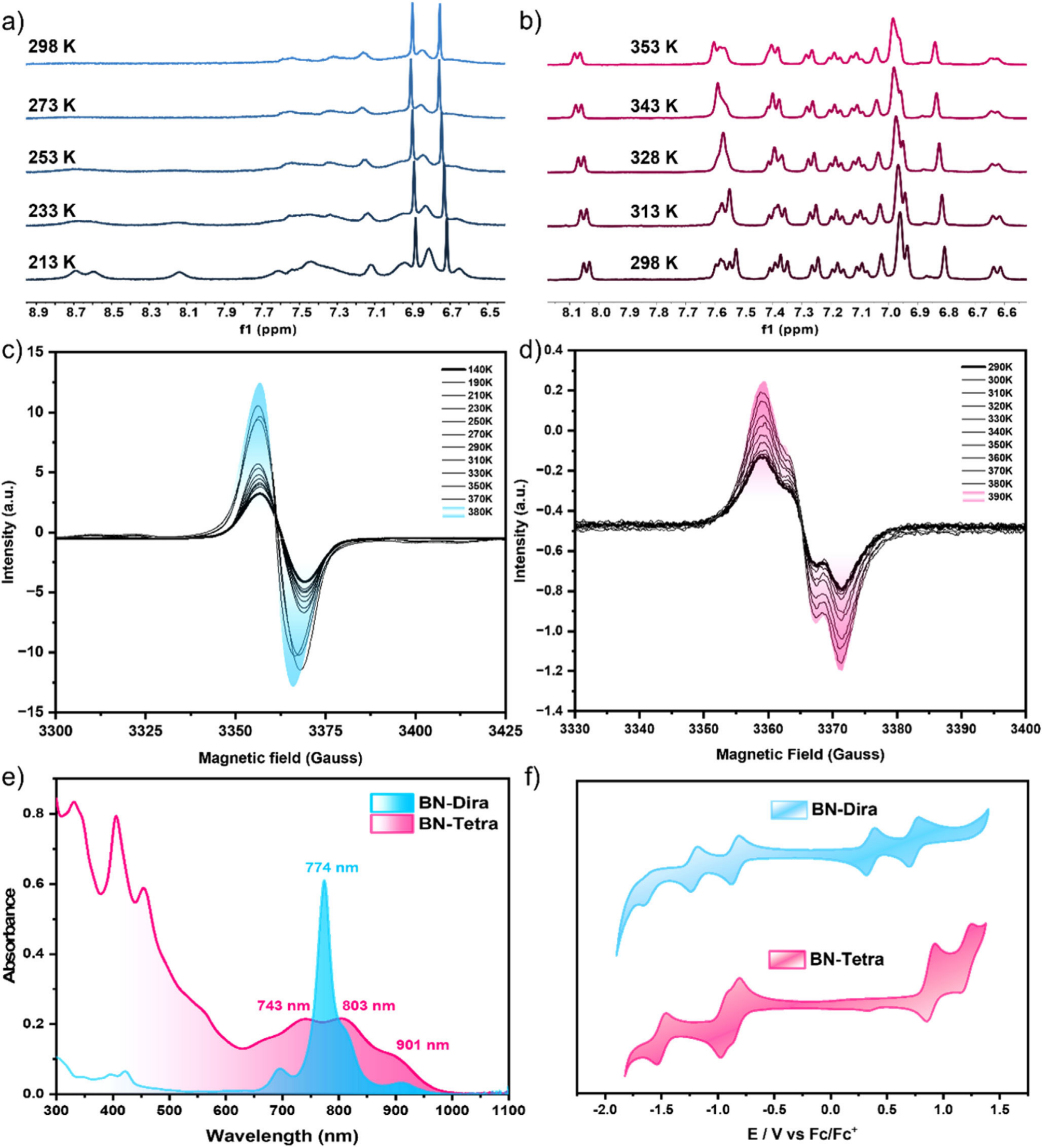
VT‐^1^H‐NMR spectra of (a) **BN‐Dira** and (b) **BN‐Tetra** in the aromatic region. VT‐EPR spectra of the microcrystalline samples of (c) **BN‐Dira** and (d) **BN‐Tetra**. (e) Absorption spectra of two PHs in dichloromethane solution (10 µM). (f) Cyclic voltammetry measurements of two PHs.

The variable‐temperature (VT) ^1^H‐NMR spectra of **BN‐Dira** (Figures 4a and S31) exhibited significant signal broadening with temperature, indicating a thermally promoted increase in the population of the paramagnetic triplet species. In contrast, the VT‑^1^H NMR spectra of **BN‑Tetra** show only minor broadening (Figures 4b and S32). Accordingly, to further confirm the magnetic properties of both compounds, VT‑EPR measurements were further investigated. VT‑EPR results reveal that the EPR signals of both **BN‑Dira** (Figure 4c) and **BN‑Tetra** (Figure 4d) intensify with increasing temperature, a hallmark of an open‑shell singlet ground state. These observations are consistent with computed biradical and tetraradical indices (**BN‑Dira**: *y*_0_  =  0.74; **BN‑Tetra**: *y*_0_  =  0.43, *y*_1_  =  0.30) and DFT‑calculated singlet–triplet energy gaps, in which the singlet ground state lies 3.76 kcal·mol^−^^1^ below the triplet state for **BN‑Dira** and 0.82 kcal·mol^−^^1^ below for **BN‑Tetra**, collectively confirming their singlet ground states. To further elucidate the effect of B–N incorporation, computational studies were performed on **BN‐Dira** and **BN‐Tetra** to enable a comprehensive comparison with their all‐carbon analogs **C‐Dira** and **C‐Tetra** (Figure S36, Table S3), respectively. As shown in Figure S36, the spin density in **C‐Dira** and **C‐Tetra** is uniformly delocalized across the molecular skeleton, in contrast to that observed for **BN‐Dira** and **BN‐Tetra**, respectively. The *y*_0_ value for **C‐Dira** is slightly larger than that of **BN‐Dira**, while its calculated HOSO–LUSO energy gap is significantly smaller. Compared with **BN‐Tetra**, **C‐Tetra** exhibits larger *y*_0_ and *y*_1_ values but a similar HOSO–LUSO energy gap. The higher radical index (*y*_0_ and *y*_1_) of **C‐Dira** and **C‐Tetra** are likely attributable to the stronger conjugation effect of the C═C double bonds relative to the B─N bonds. The trends in the values of **BN‐Dira** and **BN‐Tetra** with respect to their all carbon counterparts suggest that the introduction of B─N units provide an effective means to modulate the spin density, radical index and the HOSO–LUSO energy gap.

In addition, the photophysical properties of **BN‐Dira** and **BN‐Tetra** were examined (Figure 4e). The absorption spectrum revealed that **BN‐Dira** exhibited a broad absorption range extending to 980 nm. Notably, **BN‑Dira** shows its maximum *ε* of 137 346 M^−^^1^·cm^−^^1^ at 774 nm. At the irradiation wavelength used for PTT (808 nm), the *ε* value is 45 200 M^−^^1^·cm^−^^1^ (approximately one‑third of the peak value). This value is comparable to that of **BN‑Tetra** at 808 nm (42 800 M^−^^1^·cm^−^^1^). Nevertheless, the high PCE of **BN‑Dira** (83.6%) arises from efficient non‑radiative decay pathways in addition to absorption strength, and the *ε* at 808 nm is still sufficient to generate rapid heating. By monitoring the absorption decay at 774 nm, the half‐life time (*t*_1/2_) of **BN‐Dira** was determined to be 27 days in DCM solution under ambient condition (Figure S34). Additionally, the absorption of **BN‐Tetra** showed negligible change after one month of monitoring (Figure S34), demonstrating its high stability under ambient conditions. Furthermore, cyclic voltammetry (CV) measurements of **BN‐Dira** revealed two reversible oxidation waves and three reduction waves. **BN‐Tetra** displayed two reversible oxidation behaviors and three reversible reduction processes. The HOSO energy levels of **BN‐Dira** and **BN‐Tetra** were calculated to be −5.06 and −5.67 eV, respectively. The LUSO energy level of −4.03 eV in **BN‐Dira** and −4.21 eV in **BN‐Tetra** rendered their energy gap determined to be 1.03 and 1.46 eV, respectively (Figure 4f).

The combination of high absorption intensity in the NIR region positions these two open‐shell BN‐doped PHs as ideal photothermal conversion materials for PTT applications. To enhance the water solubility and prolong the blood circulation of **BN‐Dira** and **BN‐Tetra** in tissues for PTT, **BN‐Dira** and **BN‐Tetra** were coated with an amphiphilic polymer, 1,2‐distearoyl‐sn‐glycero‐3‐phosphoethanolamine‐poly(ethylene glycol) (DSPE‐PEG2000), to form water‐dispersible nanoparticles (**BN‐Dira NPs** and **BN‐Tetra NPs**, Figure 1b) through a conventional nanoprecipitation method [56]. Transmission electron microscopy (TEM) characterization revealed that both **BN‐Dira NPs** and **BN‐Tetra NPs** possess the homogeneous spherical morphology with good monodispersity (Figures S37b and S37e). The hydrodynamic diameter of **BN‐Dira NPs** and **BN‐Tetra NPs**, as determined by dynamic light scattering (DLS), were 103.4 and 218.7 nm, respectively (Figures S37c and S37f). Moreover, the encapsulated nanoparticles retained the absorption profiles of the two parent PHs [57]. For **BN‐Dira NPs**, it still retained strong absorption at 774 nm, a key factor underlying its high PCE. Notably, after encapsulation, both **BN‑Dira NPs** and **BN‑Tetra NPs** show a bathochromic shift and broadened absorption that extends into the NIR‑II region (Figure S37g,h), suggesting potential for deeper tissue penetration in future NIR‑II PTT applications. However, the present study focuses on 808 nm (NIR‑I) irradiation, and systematic evaluation of NIR‑II performance is beyond the current scope (Figure S37). For **BN‐Dira NPs**, the absorption spectrum in double distilled water (ddH_2_O) was redshifted and broadened relative to that in dichloromethane (Figure S37g). Similar to **BN‐Dira NPs**, a comparable redshifted was also observed in the absorption spectrum of **BN‐Tetra NPs** in ddH_2_O (Figure S37h).

**FIGURE 5:**
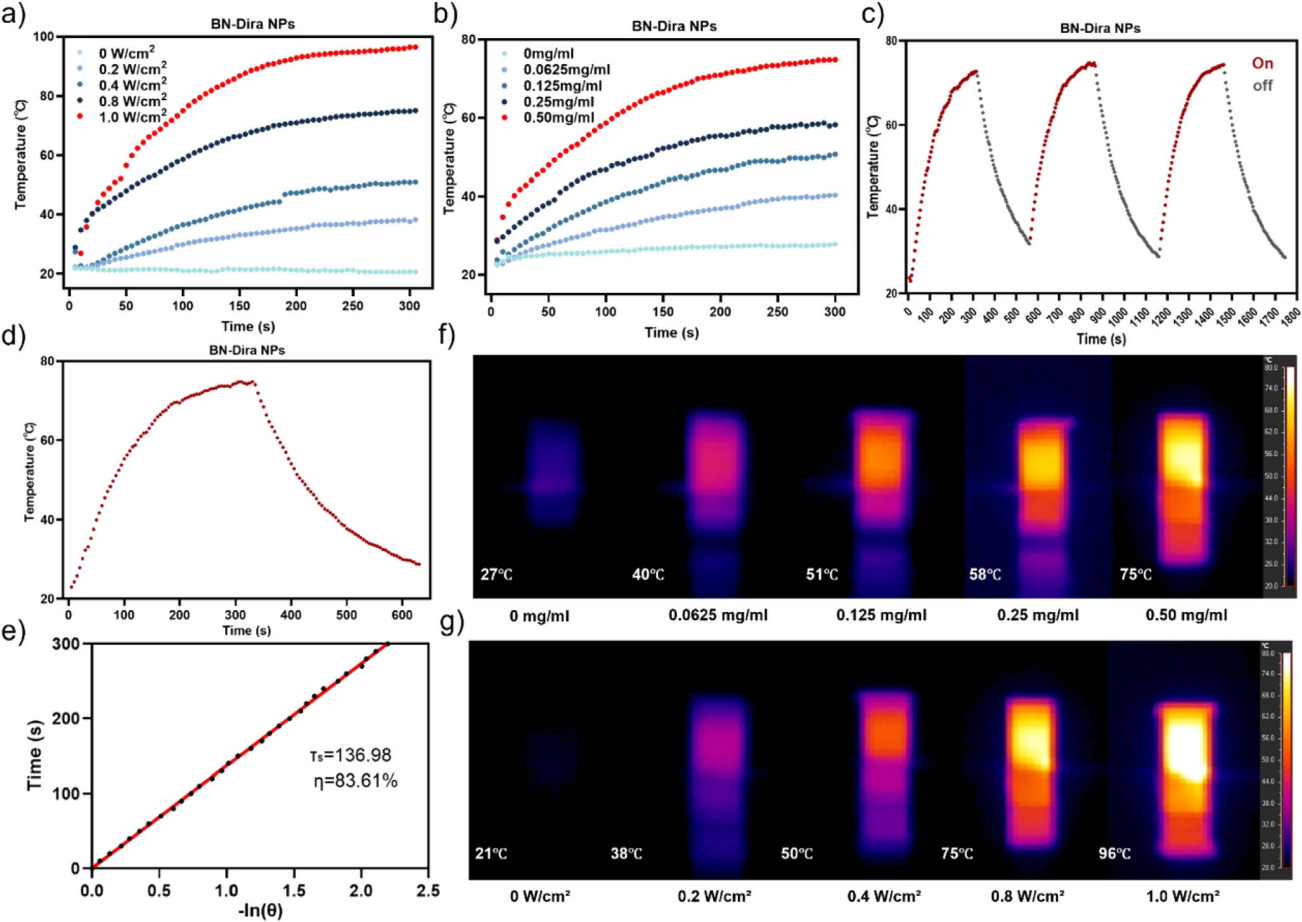
(a) Temperature–time profiles of **BN‐Dira NPs** (0.50 mg/mL) under 808 nm laser irradiation with varying power. (b) Temperature–time profiles of **BN‐Dira NPs** at different concentrations under 808 nm laser (0.8 W/cm^2^) irradiation. (c) Photothermal stability monitoring of **BN‐Dira NPs** (0.50 mg/mL) under 808 nm laser irradiation (0.8 W/cm^2^). (d) Temperature–time profile of **BN‐Dira NPs** aqueous solution (0.50 mg/mL) after 5 min of 808 nm laser (0.8 W/cm^2^) irradiation and subsequent cessation. (e) Calculation of photothermal conversion efficiency of **BN‐Dira NPs**. (f) Infrared thermal imaging of **BN‐Dira NPs** at various concentrations under 808 nm laser irradiation (0.8 W/cm^2^). (g) Infrared thermal imaging of **BN‐Dira NPs** (0.50 mg/mL) under 808 nm laser irradiation at different power levels.

The photothermal conversion capability of **BN‐Dira NPs** and **BN‐Tetra NPs** was investigated by exposing them to laser irradiation [58]. The temperature changes resulting from the irradiation of **BN‐Dira NPs** (0.5 mg/mL) with an 808 nm laser with varying power densities were recorded. A proportional relationship was observed, where the final solution temperature increased with the irradiation power at a fixed duration. Under 808 nm laser irradiation at 1.0 W/cm^2^ for 5 min, the solution temperature of **BN‐Dira NPs** rapidly increased to 96°C (Figure 5a). Similarly, **BN‐Dira NPs** also showed a concentration‐dependent temperature increase under 808 nm laser irradiation at 0.8 W/cm^2^ (Figure 5b). The excellent photothermal ability of **BN‐Dira NPs** was further demonstrated by the infrared thermal imaging (Figure 5f,g). Figure 5d showed the temperature–time curve for a solution of **BN‐Dira NPs** under 808 nm laser (0.8 W/cm^2^) irradiation and subsequent cessation. The linear relationship between the cooling time (*t*) and the negative natural logarithm of the driving force temperature (ln*θ*) provided a time constant (*τ*) of 136.98. Accordingly, the PCE of **BN‐Dira NPs** was estimated to be 83.6% (Figure 5e). Furthermore, the reproducibility of the photothermal effect was confirmed across three consecutive laser on/off cycles at 808 nm laser (0.8 W/cm^2^), demonstrating the relatively good thermal stability and photostability of **BN‐Dira NPs** (Figure 5c). The photothermal behavior of **BN‐Tetra NPs** was studied as well, demonstrating the comparable photothermal conversion capability and photostability (Figure S39). Compared to **BN‐Dira NPs**, **BN‐Tetra NPs** exhibited a lower PCE value of 69.3% (Figure S39f), which is attributed to its reduced absorption intensity in the NIR region (Figure 4e).

### 2.1 In Vitro and In Vivo Photothermal Effect

**FIGURE 6:**
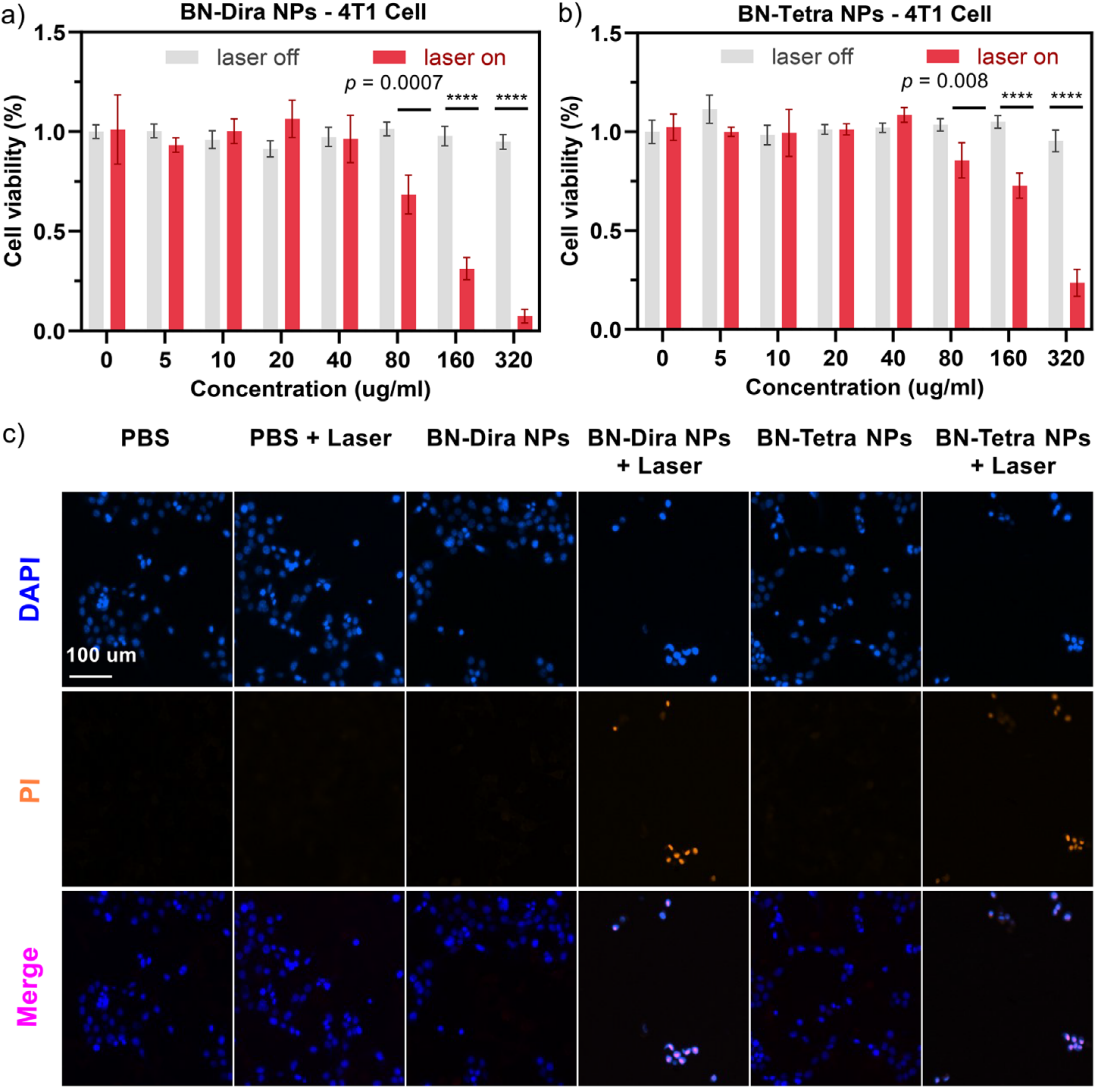
(a) Cytotoxicity of **BN‐Dira NPs** and (b) **BN‐Tetra NPs** toward 4T1 cells under 808 nm laser irradiation (1.0 W/cm^2^), and (c) DAPI/PI staining images of 4T1 cells subjected to different treatments. The data are shown as the mean ± SD from a representative experiment of two to three independent experiments with *n* = 4 biologically independent samples per group (a, b). Statistical significance was calculated via unpaired Student's *t* test (two tailed) comparing laser off vs. laser on at the same material concentration, with **** indicating *p* < 0.0001.

The compelling photothermal properties, including excellent photothermal stability and high PCE, motivated us to further investigate the photothermal effect on tumor cancers. Murine breast cancer (4T1) cells were selected as a model to test the photothermal ablation effect of **BN‐Dira NPs** and **BN‐Tetra NPs**. In the absence of laser irradiation, treatment with **BN‐Dira NPs** and **BN‐Tetra NPs** resulted in no significant reduction in cell viability, indicating their low dark toxicity (Figure 6a,b). However, upon 808 nm laser irradiation (1.0 W/cm^2^), the viabilities of 4T1 cells dramatically decreased to 7.4% ± 3.4% with **BN‐Dira NPs** (320 ug/mL), suggesting its intense photoinduced cytotoxicity. A pronounced antitumor effect was observed in 4T1 cells following treatment with **BN‐Tetra NPs** under 808 nm laser irradiation (Figure 6b). Subsequently, to assess photothermal ablation efficacy, 4T1 cells treated with **BN‐Dira NPs** and **BN‐Tetra NPs** were stained with 4',6‐diamidino‐2‐phenylindole (DAPI) and propidium iodide (PI). In all control groups, “**BN‐Dira NPs**” (**BN‐Dira NPs** incubated without laser irradiation), “**phosphate‐buffered saline (PBS)**” (**PBS** incubated), and “**PBS** + Laser” (**PBS** incubated with a 5 min laser irradiation), 4T1 cells presented uniform blue DAPI fluorescence with no significant differences between groups (Figure 6c). In contrast, treatment with “**BN‐Dira NPs** + Laser” (**BN‐Dira NPs** incubated with 5 min laser irradiation), 4T1 cells exhibited distinct red PI fluorescence, which was absent in all reference groups (Figure 6c). A comparable photothermal ablation effect was observed with the “**BN‐Tetra NPs** + Laser” group (**BN‐Tetra NPs** incubated with 5 min laser irradiation), also showing strong red PI fluorescence (Figure 6c). Additionally, the potent photoablation efficacy of both **BN‐Dira NPs** and **BN‐Tetra NPs** was further validated in B16 cells, confirming their excellent biocompatibility and robust light‐induced cytotoxicity (Figure S40).

**FIGURE 7:**
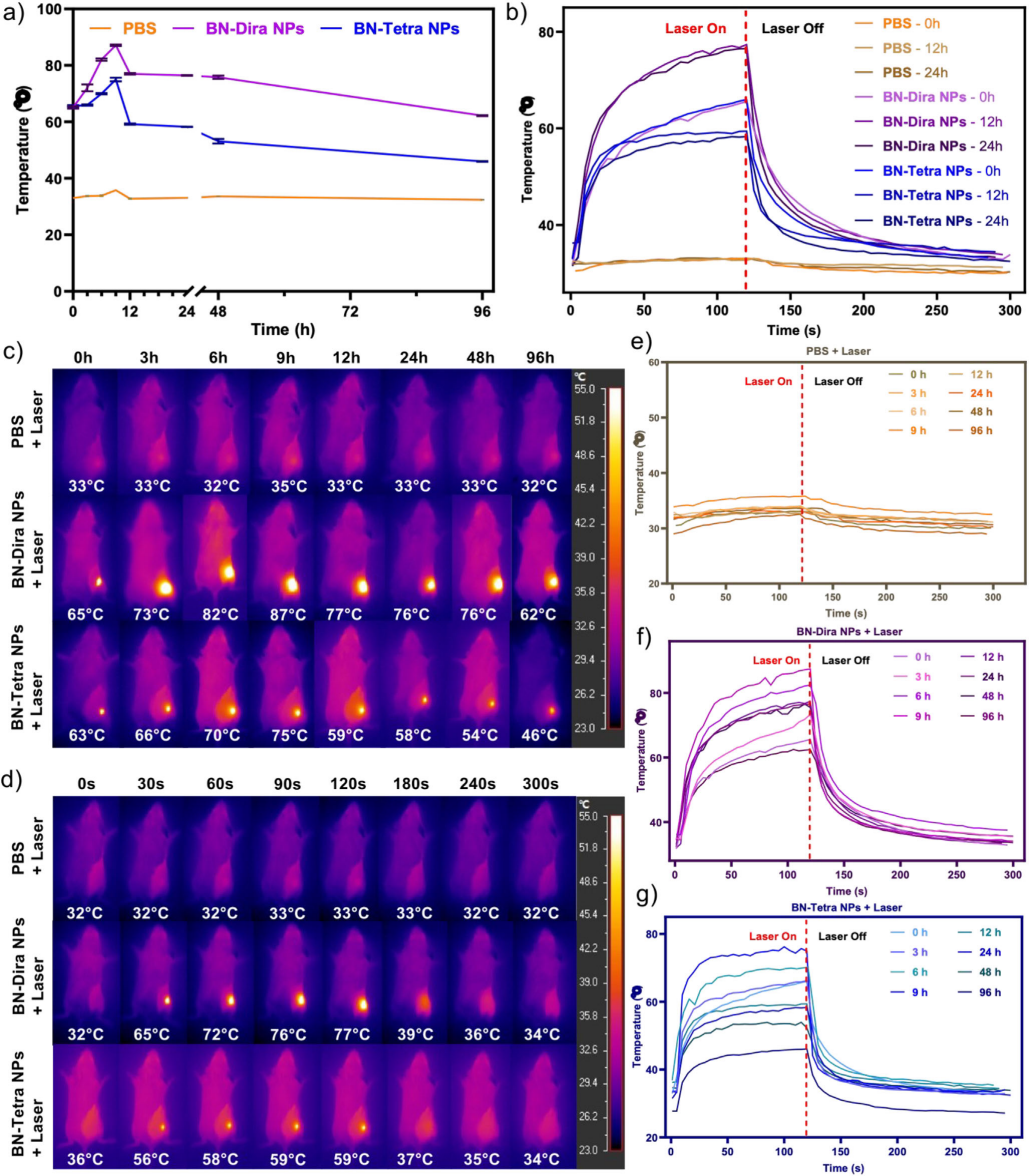
(a) Temperature change curves of tumor regions in mice under 808 nm laser irradiation (1.0 W/cm^2^) after different treatments over 96 h. (b) Temperature change curves of tumor regions in mice under 808 nm laser irradiation (1.0 W/cm^2^) after different treatments, monitored continuously for 5 min. (c) Photothermal imaging of tumor regions in mice under 808 nm laser irradiation (1.0 W/cm^2^) after different treatments over 96 h. (d) Photothermal imaging of tumor regions in mice under 808 nm laser irradiation (1.0 W/cm^2^) after different treatments, monitored continuously for 5 min. (e) Temperature change curve of tumor regions in mice injected with PBS under 808 nm laser irradiation (1.0 W/cm^2^). (f) Temperature change curve of tumor regions in mice injected with **BN‐Dira NPs** under 808 nm laser irradiation (1.0 W/cm^2^). (g) Temperature change curve of tumor regions in mice injected with **BN‐Tetra NPs** under 808 nm laser irradiation (1.0 W/cm^2^).

**FIGURE 8:**
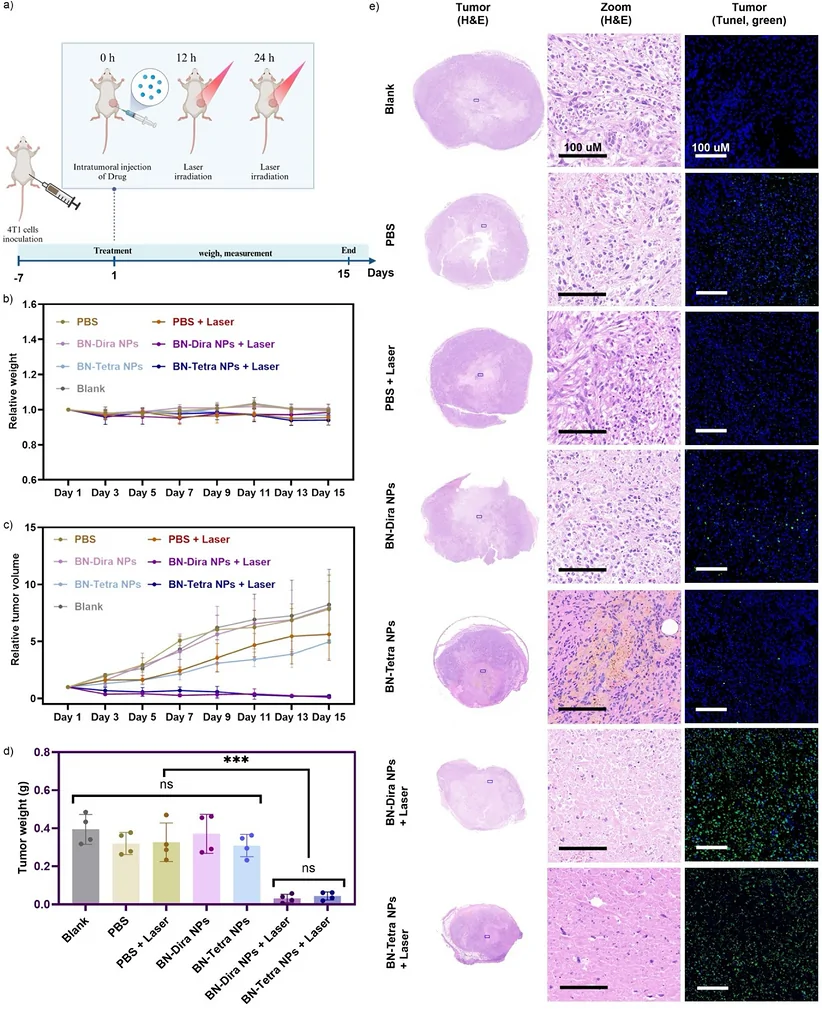
(a) Schematic diagram of the experimental design for photothermal therapy efficacy validation in tumor‐bearing mouse models. (b) Trends in relative body weight of mice. (c) Trends in relative tumor volume of mice. (d) Tumor weight in different treatment groups. (e) HE staining and TUNEL staining images (green) of tumors in different treatment group. The data are shown as the mean ± SD from a representative experiment with *n* = 4 biologically independent samples per group (b to d). Statistical significance was calculated via one‐way ANOVA in (d) with Bonferroni multiple comparisons posttest, where ns indicates no statistical significance and *** indicates *p* < 0.001.

The successful in vitro photothermal performance of **BN‐Dira NPs** and **BN‐Tetra NPs** prompted us to evaluate their in vivo photoablation efficacy in 4TI cells‐bearing mice. Three groups of 4TI cells‐bearing mice were intratumorally injected with **PBS**, **BN‐Dira NPs** and **BN‐Tetra NPs**, respectively (Figure 7). The temperature profiles of tumor regions in three groups under 808 laser irradiation (1.0 W/cm^2^) were recorded over a 96‐h period (Figure 7a). Under laser irradiation, mice injected with **PBS** showed no temperature change (32°C–35°C), while a rapid heating phase followed by gradually cooling was observed in mice injected with **BN‐Dira NPs** or **BN‐Tetra NPs**. At 3 h post‐injection, significant heat generation at the tumor site led to a sustained high temperature. Infrared thermal imaging clearly revealed distinct thermal profiles at the tumor site over the 96‐h period following different treatments under 808 nm laser irradiation (1.0 W/cm^2^) (Figure 7c). Specifically, the temperature change induced by laser on–off cycling was monitored continuously over a 5‐min period. Driven by irradiation of 2 min, the temperature in mice injected with **BN‐Dira NPs** rapidly increased to 77°C and then decreased to 34°C within 3 min after the laser was turned off (Figure 7d). The photothermal effect induced by laser cycling remained pronounced for 24 h in mice treated with **BN‐Dira NPs** or **BN‐Tetra NPs**. Remarkably, a significant response was still detectable for **BN‐Dira NPs** even 96 h post‐injection (Figure 7f,g). Infrared thermal images of the tumor regions provided direct visual confirmation of the laser‐induced temperature increase in the treated mice (Figure 7c). Furthermore, to evaluate the efficacy of PTT, tumor‐bearing mice were randomly assigned to seven treatment groups, and tumor volumes were monitored over a 15‐day period (Figure 8a). The tumor volumes in the controll groups increased steadily, while the growth was significantly suppressed in the treated groups over 15‐day period (Figure 8c). Upon completion of the 15‐day treatment regimen, the tumors were dissected, and their weights were determined accordingly. The significant reduction in tumor weight in the treated groups confirms the therapeutic efficacy of **BN‐Dira NPs**‐ and **BN‐Tetra NPs**‐mediated PTT (Figure 8d). Histological analysis further corroborated the potent antitumor efficacy of **BN‐Dira NPs** and **BN‐Tetra NPs**, as evidenced by clear necrotic areas in hematoxylin and eosin (H and E) stains and strong green fluorescence in terminal deoxynucleotidyl transferase dUTP nick‐end labeling (TUNEL) assays (Figure 8e). The body weights of mice in all groups were monitored over 15 days, there is no significant fluctuation in the treated groups compared to controls (Figure 8b), supporting a favorable biosafety profile for **BN‐Dira NPs** and **BN‐Tetra NPs**. Furthermore, H and E histological analysis of major organs of mice, including the heart, liver, spleen, lung, and kidney, were conducted (Figure S41). Compared to the control group, no significant histopathological alterations were observed, which further firmly confirmed the high biosafety of **BN‐Dira NPs** and **BN‐Tetra NPs**.

## 3 Conclusion

In summary, we precisely engineered two novel and stable open‐shell PHs (**BN‐Dira** and **BN‐Tetra**) containing 1,2‐BN units. This work provides the first example of a bonded BN‐doped tetraradicaloid (**BN‐Tetra**) with excellent ambient stability. The high PCE (up to 83.6%) combined with strong NIR light absorption in **BN‐Dira** and **BN‐Tetra** shaped them as promising candidates for PTT. Especially, the PCE of **BN‐Dira** is 3.5 times that of the previously reported 1,4‐BN‐doped PH. The efficiency in vitro PTT performance on 4T1 cancer cells of **BN‐Dira** and **BN‐Tetra NPs** validated their exciting role as PTAs. The subsequent in vivo trials in murine models demonstrated tumor eradication with reliable biocompatibility. By harnessing the synergistic effects of precise radical‐ and BN‐functionalization, along with the inherent advantages of PHs, this study outlines a protocol for designing efficient, safe, and adaptable next‐generation PTAs, representing a significant step toward personalized oncology.

## Author Contributions

**Peiyun Zeng**: methodology, data curation, & formal analysis. **Ziqi Zhuang**: data curation. **Huan Luo**: investigation, supervision, formal analysis, writing – original draft, & writing – review & editing. **Yubin Fu**: methodology. **Peiyu Wang**: data curation. **Yating Zheng**: data curation. **Gang Liu**: resources, supervision, formal analysis, & writing – review & editing. **Junzhi Liu**: conceptualization, formal analysis, supervision, resources, writing – original draft, & writing – review & editing.

## Conflicts of Interest

The authors declare no conflicts of interest.

## Supporting information




**Supporting File 1**: anie73561‐sup‐0001‐SuppMat.docx.

## Data Availability

The data that support the findings of this study are available in the Supporting Information of this article.
