# Supplementary material for: Engineering BN‐Doped Open‐Shell Polycyclic Hydrocarbons for Enhanced Photothermal Conversion and Therapy
Source: Angew Chem Int Ed Engl. 2026 Jul 11;65(38):e9715423. doi: 10.1002/anie.9715423 (PMC13573109; doi:10.1002/anie.9715423)
Supplement: Supplementary file 1 [file ANIE-65-e9715423-s001.docx]

**Supporting Information**

**Engineering BN-Doped Open-Shell Polycyclic Hydrocarbons for Enhanced Photothermal Conversion and Therapy**

Peiyun Zeng^1,2^, Ziqi Zhuang^3^, Huan Luo^1,2*^, Yubin Fu^4^, Peiyu Wang^3^, Yating Zheng^3^, Gang Liu^3*^, Junzhi Liu^1,5,6*^

1, Department of Chemistry, The University of Hong Kong, Pokfulam Road, Hong Kong 999077, China.

2, Chemistry and Chemical Engineering Guangdong Laboratory, Shantou 515031, China.

3, State Key Laboratory of Vaccines for Infectious Diseases, Center for Molecular Imaging and Translational Medicine, Xiang An Biomedicine Laboratory, National Innovation Platform for Industry-Education Integration in Vaccine Research, School of Public Health, Xiamen University, Xiamen 361102, China.

4, School of Energy and Environment, Southeast University, Nanjing 211189, China.

5, State Key Laboratory of Synthetic Chemistry, HKU-CAS Joint Laboratory on New Materials and Shanghai-Hong Kong Joint Laboratory on Chemical Synthesis, The University of Hong Kong, Pokfulam Road, Hong Kong 999077, China.

6, Materials Innovation Institute for Life Sciences and Energy (MILES), HKU-SIRI, Shenzhen 518045, China.

Correspondence to: [lhuan@hku.hk](mailto:lhuan@hku.hk); [gangliu.cmitm@xmu.edu.cn](mailto:gangliu.cmitm@xmu.edu.cn); [juliu@hku.hk](mailto:juliu@hku.hk)

**Contents**

**S1. Materials synthesis and characterization.**

**S2. Crystallographic data.**

**S3. VT- 1HNMR spectra.**

**S4. Proposed resonance structures**

**S5. Optical properties.**

**S6. Theoretical calculations**

**S7. DLS and absorption of nanoparticles**

**S8. Theoretical calculations.**

**S9. Reference.**

**S1. Materials synthesis and characterization.**

1.1 **General Remarks**

Materials and reagents. Commercially available solvents and reagents were used without further purification unless otherwise mentioned. Dry solvents were at HPLC level.

All NMR spectra were acquired on the Avance DRX Bruker 400 and 500 MHz FTNMR Spectrometer. The mass spectrometry analysis was performed on a Bruker Autoflex Speed MALDI TOF MS (Bruker Daltonics, Bremen, Germany) using dicyanobenzophenone as matrix and a Bruker Q-Tof Maxis II mass spectrometer. UV-vis-NIR absorption spectra were recorded on an Agilent Cary 69 UV-vis-NIR spectrophotometer using 10 mm optical-path quartz cell at room temperature. Cyclic voltammetry measurements were analyzed on a CHI660E (CH Instruments, China) in a three-electrode cell. Electrolyte solutions were prepared by dissolving 0.1 M tetrabutylammoniumhexafluorophosphate (*n*Bu_4_NPF_6_) and samples in anhydrous tetrahydrofuran (THF) and the experiments were performed with a scan rate of 50 mVs^-1^ at room temperature in nitrogen atmosphere. A glassy carbon and a Pt-sheet were used as working and counter electrode respectively. A silver wire was used as pseudo reference electrode. All potentials are given against ferrocenium/ferrocene (Fc^+^/Fc) redox couple.

**1.2 Synthetic routes and detailed procedures**

**Scheme S1.** Synthesis of **BN-Dira** and **BN-Tetra**. Reaction conditions: a: HI, toluene, 110 ⁰C, 12 h, 50%; b: BBr_3_, MesMgBr, toluene, 110 ⁰C, 12 h, 30%; c: (2-Formylphenyl)boronic acid, Pd(PPh_3_)_4_, K_2_CO_3_, dioxane/H_2_O, 24 h, 50%; d: 1) MesMgBr, THF, 2) BF_3_•OEt_2_, DCM, 80%; e: *t*-BuOK, THF, 80%; f: 2-(4,4,5,5-Tetramethyl-1,3,2-dioxaborolan-2-yl)isophthalaldehyde, Pd(OAc)_2_, SPhos, K_3_PO_4_, dioxane/H_2_O, 90 ⁰C, 12 h, 40%.

**1.2.1 Synthesis of compound 4**

To a solution of 1*H*-indole (2.49 g, 21.27 mmol) and 5-bromo-1-naphthaldehyde (5 g, 21.27 mmol) in toluene (100 mL), hydriodic acid (HI) (57%, 1.22 mL, 10.63 mmol) was slowly added at room temperature. The reaction mixture was stirred at 110°C for 14 hours. After cooling down to room temperature, the mixture was poured into water and extracted with ethyl acetate. The combined organic layer was washed with sodium sulfite solution and dried over sodium sulfate (Na_2_SO_4_). The solvents were removed under reduced pressure, and the residue was purified with column chromatography (silica gel, CH_2_Cl_2_/hexane (1/20, v/v) as eluent) to give compound **4** (4.1 g, 29%) as a pale yellow solid.

^1^H NMR (400 MHz, CDCl_3_) *δ* 8.66 (d, *J* = 8.1 Hz, 1H), 7.85 – 8.06 (m, 3H), 7.81 – 7.61 (m, 2H), 7.30 – 7.14 (m, 3H), 6.81 (s, 2H).

^13^C NMR (101 MHz, CDCl_3_) *δ* 140.93, 134.99, 134.62, 133.41, 132.84, 130.78, 129.33, 128.20, 127.75, 127.16, 126.21, 125.87, 123.51, 123.00, 122.16, 121.54, 118.90, 114.87, 110.32.

MS (MALDI): m/z [M] calcd. for 666.0127, found 666.0341.

**Figure S1.** ^1^H NMR spectrum of compound **4** in CDCl_3_ at 298 K.

**Figure S2.** ^13^C NMR spectrum of compound **4** in CDCl_3_ at 298 K.

**Figure S3.** Mass spectrum of compound **4**.

**1.2.2 Synthesis of compound 3**

To an ice cooled solution of **4** (700 mg, 1.05 mmol) in 12 mL dry toluene was added dropwise BBr_3_ (1.0 mL, 10.5 mmol). After stirring for 1 h, the solution warmed to room temperature and stirred for 1 h. The resulting mixture was stirred at 110^o^C for 6 h. After being cooled to room temperature, 2-mesitylmagnesium bromide (MesMgBr, 10.5 mL, 10.5 mmol, 1.0 M in THF) was added to the mixture and stirred at room temperature for another 1 h. The reaction was quenched with 10 mL H_2_O. The resulting mixture was extracted with ethyl acetate, washed by water, dried over anhydrous Na_2_SO_4_. The solution was concentrated under reduced pressure, and the residue was purified by flash column chromatography using CH_2_Cl_2_/hexane (1/10, v/v) as eluent to give **3** as a bright yellow solid (550 mg, yield: 57%).

^1^H NMR (500 MHz, CDCl_3_) *δ* 8.64 (d, *J* = 8.5 Hz, 1H), 8.49 (d, *J* = 8.5 Hz, 1H), 8.08 (d, *J* = 8.5 Hz, 1H), 7.95 (d, *J* = 7.4 Hz, 1H), 7.22 (t, *J* = 8.0 Hz, 1H), 7.17 – 7.10 (m, 3H), 7.05 (s, 1H), 6.91 (t, *J* = 7.1 Hz, 1H), 6.83 (d, *J* = 8.0 Hz, 1H), 2.52 (s, 3H), 2.29 (s, 3H), 2.00 (s, 3H).

^13^C NMR (101 MHz, CDCl_3_) *δ* 143.85, 139.88, 139.45, 138.41, 138.37, 138.04, 135.48 (C-B), 134.94 (C-B), 133.85, 133.51, 131.56, 131.37, 130.46, 128.85, 127.86, 127.79, 127.17, 126.79, 126.57, 125.11, 123.78, 123.08, 122.72, 116.96, 115.07, 22.96, 22.53, 21.56.

MS (MALDI) m/z [M] calcd. for 922.1742, found 922.2188.

**Figure S4.** ^1^H NMR spectrum of compound **3** in CDCl_3_ at 298 K.

**Figure S5**. ^13^C NMR spectrum of compound **3** in CDCl_3_ at 298 K.

**Figure S6.** Mass spectrum of compound **3**.

**1.2.3 Synthesis of compound 2a**

In a Schlenk tube, a mixture of **3** (300 mg, 0.33 mmol), (2-formylphenyl)boronic acid (146.3 mg, 0.98 mmol), tetrakis(triphenylphosphine)palladium(0) (Pd(PPh_3_)_4_, 37.6 mg, 0.03 mmol) and potassium carbonate (269.7 mg, 1.95 mmol) was dissolved in 1,4-dioxane (30 mL) and water (4 mL) under nitrogen. The reaction mixture was stirred at 95 ^o^C for 12 h. After cooling down to room temperature, the mixture was poured into water and extracted with ethyl acetate three times. The combined organic layer was washed with water and dried over Na_2_SO_4_. The solvents were removed under reduced pressure, and the residue was purified with column chromatography (silica gel, CH_2_Cl_2_/hexane (1/15, v/v) as eluent) to give compound **2** (225 mg, 81%) as a bright yellow solid.

^1^H NMR (400 MHz, C_2_D_2_Cl_4_) *δ* 10.11 (s, 1H), 9.68 (s, 1H), 8.81 (d, *J* = 8.6 Hz, 2H), 8.22 (ddd, *J* = 10.1, 8.2, 1.3 Hz, 2H), 7.95 (ddd, *J* = 8.1, 5.3, 2.6 Hz, 2H), 7.86 (d, *J* = 4.0 Hz, 3H), 7.78 (d, *J* = 8.5 Hz, 1H), 7.73 (td, *J* = 7.1, 5.7, 3.3 Hz, 4H), 7.65 (dt, *J* = 7.3, 1.5 Hz, 2H), 7.54 (ddd, *J* = 10.1, 8.6, 6.9 Hz, 2H), 7.26 – 7.18 (m, 4H), 7.14 (d, *J* = 4.1 Hz, 2H), 7.07 – 7.04 (m, 2H), 7.03 – 6.97 (m, 3H), 6.94 (d, *J* = 8.0 Hz, 1H), 2.52 (s, 6H), 2.33 (s, 6H), 2.06 (s, 6H).

^13^C NMR (126 MHz, CD_2_Cl_2_) *δ* 191.82, 191.54, 144.37, 144.09, 144.06, 144.04, 139.89, 139.85, 139.73, 139.70, 139.41, 139.37, 139.29, 139.25, 138.54, 138.51, 138.47, 138.14, 138.11, 137.97, 135.99, 135.98, 135.86, 135.62 (C-B), 135.16, 134.95, 134.55(C-B), 134.01, 133.55, 132.84, 132.81, 132.65, 132.62, 132.37, 132.07, 130.99, 130.97, 130.27, 130.11, 130.02, 129.45, 129.19, 129.17, 129.08, 129.06, 129.02, 128.94, 128.52, 128.44, 128.21, 127.82, 127.72, 127.70, 127.58, 127.42, 127.04, 127.01, 125.92, 125.70, 125.66, 125.51, 125.35, 125.29, 125.20, 125.16, 125.13, 123.94, 123.92, 123.90, 122.78, 122.68, 117.28, 117.22, 115.08, 115.06, 22.64, 22.59, 22.22, 22.20, 21.27, 21.22.

MS (MALDI) m/z [M] calcd. for 972.4074, found 972.4283.

**Figure S7.** ^1^H NMR spectrum of compound **2** in C_2_D_2_Cl_4_ at 298 K.

**Figure S8.** ^13^C NMR spectrum of compound **2** in CD_2_Cl_2_ at 298 K.

**Figure S9.** COSY spectrum of compound **2** in CDCl_3_ at 298 K.

 **Figure S10.** ROESY spectrum (region) of compound **2** in CDCl_3_ at 298 K.

**Figure S11.** Mass spectrum of compound **2**.

**1.2.4 Synthesis of compound 1**

Compound **2** (150 mg, 0.15 mmol) was added into THF (20 mL) in a dry Schlenk tube under nitrogen, and a solution of MesMgBr in THF (1.54 mL, 1 M in THF, 1.54 mmol) was slowly added at room temperature. The mixture was stirred for 30 minutes, then quenched by slow addition of water. After filtering, the filtrate was poured into water and extracted three times with ethyl acetate. The combined organic phase was dried over Na_2_SO_4_. The solvent was removed under reduced pressure. The obtained solid was transferred to a dry Schlenk flask and then dry CH_2_Cl_2_ (15 mL) was then added under nitrogen. A solution of BF_3_∙Et_2_O (0.19 mL, 1.54 mmol) was added dropwise at room temperature. The mixture was stirred for 30 minutes and quenched by slow addition of methanol, the mixture was poured into water and extracted with CH_2_Cl_2_ three times, the combined organic layer was washed with water and dried over Na_2_SO_4_. The solvents were removed under reduced pressure, and the residue was purified with column chromatography (silica gel, CH_2_Cl_2_/hexane (1/20, v/v) as eluent) to give compound **1** as a bright yellow solid. (128 mg, 71% in two steps).

^1^H NMR (400 MHz, CD_2_Cl_2_) *δ* 8.54 (d, *J* = 8.5 Hz, 1H), 8.31 (d, *J* = 7.4 Hz, 1H), 8.27 (d, *J* = 7.9 Hz, 1H), 7.38 – 7.50 (m, 3H), 7.27 (t, *J* = 7.5 Hz, 1H), 7.21 (d, *J* = 1.9 Hz, 1H), 7.15 (ddd, *J* = 8.4, 5.7, 2.7 Hz, 1H), 7.11 (s, 1H), 7.06 (d, *J* = 7.7 Hz, 1H), 6.99 – 6.90 (m, 3H), 6.84 (s, 1H), 6.76 (s, 1H), 6.31 (s, 1H), 2.53 (s, 3H), 2.42 (s, 3H), 2.35 (s, 3H), 2.29 (s, 3H).

^13^C NMR (101 MHz, CD_2_Cl_2_) *δ* 143.86, 140.35, 139.41, 139.35, 138.28, 138.17, 137.94, 137.33, 137.14, 136.97, 136.09, 134.62, 132.14, 130.88, 130.78, 130.67, 130.44, 130.05, 129.04, 128.15, 128.09, 127.93, 127.31, 127.17, 126.78, 126.53, 126.29, 125.03, 123.44, 123.25, 122.41, 120.47, 116.90, 114.73, 53.97, 53.70, 53.43, 53.16, 52.89, 43.36, 31.93, 29.69, 29.36, 22.69, 22.38, 22.01, 21.13, 20.58, 20.50, 20.19, 13.88.

MS (MALDI) m/z [M-H]^-^ calcd. for 1175.5666, found 1175.5767.

**Figure S12.** ^1^H NMR spectrum of compound **1** in CD_2_Cl_2_ at 298 K.

**Figure S13.** ^13^C NMR spectrum of compound **1** in CDCl_2_ at 298 K.

**Figure S14.** COSY spectrum of compound **1** in CDCl_3_ at 298 K.

**Figure S15.** ROESY spectrum of compound **1** in CDCl_3_ at 298 K.

**Figure S16.** Mass spectrum of compound **1**.

**1.2.5 Synthesis of compound BN-Dira**

Under nitrogen atmosphere, the precursor **1** (100 mg, 0.085 mmol) and potassium tert-butoxide (*t*-BuOK, 38 mg, 0.34 mmol) were added to a 25 ml flask, then dry THF (15 ml) was added and then stirred for 0.5 h. The reaction mixture was diluted in ethyl acetate and washed by water. Then the organic layer was dried over Na_2_SO_4_. The solvents were removed under reduced pressure, and the residue was purified with column chromatography (silica gel, CH_2_Cl_2_/hexane (1/20, v/v) as eluent) to give **BN-Dira** (89 mg, 89%) as a green solid.

MS (MALDI) m/z [M] calcd. for 1174.5588, found 1174.5646.

**Figure S17.** Mass spectrum of **BN-Dira**.

**1.2.6 Synthesis of compound 5**

In a Schlenk tube, a mixture of compound **3** (250 mg, 0.27 mmol), (2-formyl-1,3-phenylene)diboronic acid (212 mg, 0.81 mmol), potassium phosphate (345 mg, 1.63 mmol), palladium(II) acetate (Pd(OCOCH_3_)_2_, 6 mg, 0.027 mmol) and S-Phos (22 mg, 0.054 mmol) was dissolved in 1,4-dioxane (30 mL) and water (4 mL) under nitrogen. The reaction mixture was stirred at 95 ^o^C for 12 h. After cooling down to room temperature, the mixture was poured into water and extracted with ethyl acetate three times. The combined organic layer was washed with water and dried over Na_2_SO_4_. The solvents were removed under reduced pressure, and the residue was purified with column chromatography (silica gel, CH_2_Cl_2_/hexane (1/5, v/v) as eluent) to give compound **5** (150 mg, 54%) as a bright yellow solid.

^1^H NMR (400 MHz, CD_2_Cl_2_) *δ* 10.13 (s, 1H), 9.61 (s, 1H), 8.93 (d, *J* = 8.5 Hz, 1H), 8.47 (td, *J* = 8.0, 3.9 Hz, 2H), 7.98 (d, *J* = 8.4 Hz, 1H), 7.89 (t, *J* = 7.8 Hz, 1H), 7.76 (d, *J* = 6.9 Hz, 1H), 7.65 – 7.55 (m, 2H), 7.28 – 7.15 (m, 3H), 7.11 – 6.99 (m, 3H), 2.50 (s, 3H), 2.34 (s, 3H), 2.02 (s, 3H).

^13^C NMR (126 MHz, CD_2_Cl_2_) *δ* 190.67, 190.28, 146.34, 144.05, 139.79, 139.32, 138.58, 138.54, 138.19, 135.88, 135.66, 135.32 (C-B), 135.24, 134.84 (C-B), 133.44, 133.08, 132.65, 131.87, 130.79, 130.57, 129.93, 129.11, 128.98, 127.77, 127.74, 127.13, 125.38, 125.06, 123.93, 122.81, 117.12, 115.15, 22.61, 22.16, 21.22.

MS (MALDI) m/z [M] calcd. for 1028.3973, found 1028.4318.

**Figure S18.** ^1^H NMR spectrum of compound **5** in CD_2_Cl_2_ at 298 K.

**Figure S19.** ^13^C NMR spectrum of compound **5** in CD_2_Cl_2_ at 298 K.

**Figure S20.** Mass spectrum of compound **5**.

**1.2.7 Synthesis of compound 6**

The compound **5** (80 mg, 0.078 mmol) was added into THF (15 mL) in a dry Schlenk tube under nitrogen, and a solution of 2-mestityl magnesium bromide in THF (0.8 mL, 1 M in THF, 0.78 mmol) was slowly added at room temperature. The mixture was stirred for 30 minutes, then quenched by slow addition of water. After filtering, the filtrate was poured into water and extracted three times with ethyl acetate. The combined organic phase was dried over Na_2_SO_4_. The solvent was removed under reduced pressure. The obtained solid was transferred to a dry Schlenk flask and then dry CH_2_Cl_2_ (15 mL) was then added under nitrogen. A solution of BF_3_∙Et_2_O (0.13 mL, 1.03 mmol) was added dropwise at room temperature. The mixture was stirred for 30 minutes and quenched by slow addition of methanol, the mixture was poured into water and extracted with CH_2_Cl_2_ three times, the combined organic layer was washed with water and dried over Na_2_SO_4_. The solvents were removed under reduced pressure and the residue was purified with column chromatography (silica gel, CH_2_Cl_2_/hexane (1/20, v/v) as eluent) to give compound **6** as a bright yellow solid. (70 mg, 63% in two steps).

^1^H NMR (500 MHz, CD_2_Cl_2_) *δ* 8.43 – 8.30 (m, 2H), 7.65 (d, *J* = 6.9 Hz, 1H), 7.43 – 7.34 (m, 2H), 7.19 – 7.34 (m, 9H), 7.18 – 7.12 (m, 2H), 7.10 (s, 1H), 7.05 (d, *J* = 7.3 Hz, 3H), 6.93 – 7.02 (m, 5H), 6.88 (d, *J* = 10.7 Hz, 3H), 6.81 (s, 1H), 6.63 (s, 1H), 6.56 (s, 1H), 6.00 (s, 1H), 5.77 (s, 1H), 2.80 (s, 2H), 2.74 (s, 3H), 2.63 (s, 2H), 2.57 (d, *J* = 5.3 Hz, 5H), 2.50 (d, *J* = 12.9 Hz, 7H), 2.41 – 2.33 (m, 8H), 2.26 (s, 2H), 2.01 – 1.90 (m, 6H), 1.88 – 1.80 (m, 4H), 1.75 (d, *J* = 2.7 Hz, 3H).

^13^C NMR (126 MHz, CD_2_Cl_2_) *δ* 145.32, 144.81, 144.02, 144.00, 143.91, 143.89, 141.29, 141.21, 139.86, 139.67, 139.59, 139.32, 138.66, 138.61, 138.58, 138.47, 138.44, 138.42, 138.27, 138.22, 138.17, 138.14, 138.11, 138.06, 137.98, 137.90, 137.82, 137.69, 137.64, 137.51, 137.18, 136.45, 136.35, 136.30, 136.27, 136.19, 135.87, 135.72, 135.46, 135.36, 135.29, 134.15, 133.95, 132.77, 132.50, 131.75, 130.85, 130.80, 130.75, 130.43, 130.21, 129.38, 129.33, 129.28, 129.22, 129.10, 128.98, 128.93, 128.52, 128.43, 128.28, 128.23, 128.06, 127.93, 127.58, 127.52, 127.43, 127.32, 127.26, 127.03, 126.60, 126.50, 125.61, 125.53, 124.63, 123.51, 122.98, 122.93, 122.91, 122.47, 122.15, 122.07, 116.82, 114.95, 114.89, 114.84, 52.70, 52.62, 44.15, 43.59, 29.74, 22.79, 22.77, 22.37, 22.35, 22.00, 21.91, 21.67, 21.22, 21.17, 21.01, 20.78, 20.73, 20.63, 20.61, 20.52, 20.33, 20.31, 20.03, 18.90, 18.72, 18.70.

HR MS (MALDI) m/z [M-H]^-^ calcd. for 1435.7235, found 1435.7239.

**Figure S21.** ^1^H NMR spectrum of compound **6** in CD_2_Cl_2_ at 298 K.

**Figure S22.** ^13^C NMR spectrum of compound **6** in CD_2_Cl_2_ at 298 K.

**Figure S23.** COSY spectrum of compound **6** in CDCl_3_ at 298 K.

**Figure S24.** ROESY spectrum of compound **6** in CDCl_3_ at 298 K.

**Figure S25.** High resolution mass spectrum of compound **6**.

**1.2.8 Synthesis of compound BN-Tetra**

Under nitrogen atmosphere, the precursor **6** (36 mg, 0.025 mmol) and *t*-BuOK (23 mg, 0.20 mmol) were added to a 25 ml flask, then dry THF (10 ml) was added and then stirred for 1 h. The reaction mixture was diluted in ethyl acetate and washed by water. Then the organic layer was dried over Na_2_SO_4_. The solvents were removed under reduced pressure and the residue was purified with column chromatography (silica gel, CH_2_Cl_2_/hexane (1/20, v/v) as eluent) to give compound **BN-Tetra** (27 mg, 75%) as a brown solid.

MS (MALDI) m/z [M] calcd. for 1432.7000, found 1432.7242.

**Figure S26.** Mass spectrum of **BN-Tetra**.

**S2. Crystallographic data.**

**Table S1** X-ray crystallographic data of **BN-Dira** (CCDC 2505631).

**Table S2** X-ray crystallographic data of **BN-Tetra** (CCDC 2505632)

**Figure S27.** X-ray solid-state structures of (a) **BN-Dira** and (b) bond length in **BN-Dira**. Hydrogen atoms were omitted for clarity.

**Figure S28.** X-ray solid-state structures of (a) **BN-Tetra** and (b) bond length in **BN-Tetra**. Hydrogen atoms were omitted for clarity.

**Figure S29**. Crystal packing of **BN-Dira**. Hydrogen atoms were omitted for clarity.

**Figure S30**. Crystal packing of **BN-Tetra**. Hydrogen atoms were omitted for clarity.

**S3. VT - ^1^HNMR spectra.**

**Figure S31.** Variable-temperature ^1^H NMR spectra of **BN-Dira** in CD_2_Cl_2_ (a) from 213 K to 298 K; (b) Partially zoomed spectra in the aromatic region.

**Figure S32.** Variable-temperature ^1^H NMR spectra of **BN-Tetra** in C_2_D_2_Cl_4_ (a) from 298 K to 353 K; (b) Partially zoomed spectra in the aromatic region; (c) Partially zoomed spectra in the non-aromatic region.

**S4. Proposed resonance structures**

**Figure S33.** Proposed resonance structures of (a) **BN-Dira** and (b) **BN-Tetra**.

**S5. Optical properties_._**

**Figure S34.** Time-dependent changes of the UV-vis-NIR absorption spectra of (a) **BN-Dira** and (c) **BN-Tetra** in dichloromethane solution (concentration: 10 μM). (b) Changes of the optical density at the maximum absorption at 774 nm of **BN-Dira** under ambient conditions (black dots), linear fitting of the changes (red line).

**S6. Theoretical calculations**

All calculations were carried out using the Gaussian 09 program.^2^ Density functional theory (DFT) calculations were carried out at the UB3LYP/6-311G(d) level of theory. The optimized geometries were used for the subsequent calculations, including nucleus independent chemical shift (NICS) and anisotropy of the induced current density (ACID). NICS values were calculated GIAO-UB3LYP/6-311+g(d, 2p). ACID plots were calculated at CSGT-UB3LYP/6-31g(d) using the method developed by Herges. To assign the absorption bands observed in the UV-vis-NIR spectra of **BN-Dira** and **BN-Tetra**, time-dependent DFT calculations were performed on using Gaussian 09 program at the UB3LYP/6-311G(d) level of theory.

**Figure S35.** Calculated a) SOMO-*α* and b) SOMO-*β* of **BN-Dira**. Calculated c) SOMO-*α* and d) SOMO-*β* of **BN-Tetra**.

**Figure S36.** Molecular structures of a) **BN-Dira**, c) **BN-Tetra**, e) **C-Dira** and g) **C-Tetra**. Calculated spin density of b) **BN-Dira**, d) **BN-Tetra**, f) **C-Dira** and h) **C-Tetra**.

**Table S3.** Summary of the radical characters and HOMO-LUMO energy gaps in **BN-Dira**, **C-Dira**, **BN-Tetra** and **C-Tetra**.

| Compounds | y_0_ | y_1_ | ∆*E*_g_*^a^* (eV) | ∆*E_g_^b^* (eV) |
| --- | --- | --- | --- | --- |
| **BN-Dira** | 0.74 | / | 1.51 | 1.03 |
| **C-Dira** | 0.78 | / | 0.98 | / |
| **BN-Tetra** | 0.40 | 0.29 | 1.45 | 1.46 |
| **C-Tetra** | 0.49 | 0.36 | 1.65 | / |

**Table S4.** Selected TD-DFT (UB3LYP/6-311g (d,p)) calculated wavelength, oscillator strength and contributions of major electronic transitions of **BN-Dira**.

| **Energy (eV)** | **Wavelength (nm)** | ***f*** | **Major contribution*** |
| --- | --- | --- | --- |
| 0.8301 | 1493.61 nm | 0.00040 | Ha$\to\mathrm{La}$51.3%  Hb $\to$ Lb 48.1% |
| 1.3642 | 908.84 | 1.31660 | Ha$\to La 47.5$%  Hb $\to$ Lb 50.7% |
| 1.7723 | 699.57 | 0.00310 | Ha$\to\mathrm{La}$+1 42.3%  Hb $\to$ Lb+1 39.5% |
| 1.9811 | 625.84 | 0.00120 | Ha$\to\mathrm{La}$+1 31.6%  Ha$\to\mathrm{La}$+2 7.6%  Hb$\to\mathrm{Lb}$+1 35.4%  Hb$\to\mathrm{Lb}$+2 6.5% |
| 2.1391 | 579.61 | 0.03130 | Ha$-1 \to\mathrm{La}$ 82.6%  Hb-2 $\to\mathrm{Lb}$ 5.6% |
| 2.1545 | 575.47 | 0.03850 | Ha$-1 \to\mathrm{La}$ 75.4%  Hb-2 $\to\mathrm{Lb}$ 8.5% |
| 2.2192 | 558.69 | 0.07820 | Hb-2 $\to$Lb 26.1%  Ha-2 $\to$La 25.9%  Hb-1 $\to$Lb 9.7%  Ha $\to$La+2 5.5%  Ha-1 $\to$La 5.0% |
| 2.2518 | 550.60 | 0.00120 | Ha $\to$La+2 21.2%  Hb $\to$Lb+2 17.1%  Hb-3 $\to$Lb 10.0%  Ha-3 $\to$La 9.7%  Ha $\to$La+1 9.3%  Hb $\to$Lb+1 9.1% |
| 2.2965 | 539.88 | 0.12820 | Ha-3 $\to$La 19.0%  Hb-3 $\to$Lb 14.6%  Hb $\to$Lb+2 11.1%  Ha $\to$La+2 9.0%  Hb-2 $\to$ Lb 7.4%  Hb $\to$Lb+3 6.8% |
| 2.3659 | 524.05 | 0.01140 | Ha-2 $\to$La 34.8%  Hb-2 $\to$Lb 28.0%  Ha $\to$La+4 7.3% |

*Ha: HOMO ($\alpha$); Hb: HOMO ($\beta$); La: LUMO ($\alpha$); Lb: LUMO ($\beta$)

**Table S5.** Selected TD-DFT (UB3LYP/6-311g (d,p)) calculated wavelength, oscillator strength and contributions of major electronic transitions of **BN-Tetra**.

| **Energy (eV)** | **Wavelength (nm)** | ***f*** | **Major contribution*** |
| --- | --- | --- | --- |
| 0.6473 | 1915.41 | 0.00000 | Ha $\to$ La 38.5%  Hb $\to$ Lb 38.5%  Ha-1 $\to$ La+1 10.4%  Hb-1 $\to$ Lb+1 10.4% |
| 0.8016 | 1546.71 | 0.00000 | Ha-1 $\to$ La 24.2%  Hb-1 $\to$ Lb 24.2%  Hb $\to$ Lb+1 21.0%  Ha $\to$ La+1 21.0% |
| 1.4516 | 854.12 | 0.43470 | Hb $\to$ Lb 47.0%  Ha $\to$ La 47.0% |
| 1.6421 | 755.03 | 0.09140 | Ha $\to$ La+1 28.7%  Hb $\to$ Lb+1 28.7%  Hb-1 $\to$ Lb 18.9%  Ha-1 $\to$ La 18.9% |
| 1.7451 | 710.47 | 0.00000 | Ha $\to$ La+1 17.7%  Hb $\to$ Lb+1 17.7%  Ha-1 $\to$ La+1 10.1%  Hb-1 $\to$ Lb+1 10.1%  Ha-1 $\to$ La 8.9%  Hb-1 $\to$ Lb 8.9% |
| 1.7561 | 706.02 | 0.00000 | Ha-1 $\to$ La+1 17.8%  Hb-1 $\to$ Lb+1 17.8%  Hb-1 $\to$ Lb 10.5%  Ha-1 $\to$ La 10.5%  Hb $\to$ Lb 6.4%  Ha $\to$ La 6.4% |
| 1.7854 | 694.43 | 0.00250 | Ha-1 $\to$ La 29.1%  Hb-1 $\to$ Lb 29.1%  Hb $\to$ Lb+1 20.2%  Ha $\to$ La+1 20.2% |
| 1.7954 | 690.57 | 0.00000 | Ha-5 $\to$ La 13.3%  Hb-5 $\to$ Lb 13.3%  Hb-4 $\to$ Lb+1 12.7%  Ha-4 $\to$ La+1 12.7%  Hb-3 $\to$ Lb 6.8%  Ha-3 $\to$ La 6.8%  Ha-4 $\to$ La 6.2%  Hb-4 $\to$ Lb 6.2% |
| 1.8193 | 681.49 | 0.00000 | Ha-4 $\to$ La 16.0%  Hb-4 $\to$ Lb 16.0%  Ha-5 $\to$ La+1 9.2%  Hb-5 $\to$ Lb+1 9.2% |
| 1.9929 | 622.13 | 0.08270 | Ha-1 $\to$ La+1 44.9%  Hb-1 $\to$ Lb+1 44.9% |

*Ha: HOMO ($\alpha$); Hb: HOMO ($\beta$); La: LUMO ($\alpha$); Lb: LUMO ($\beta$)

**S7. DLS and absorption of nanoparticles**

**Figure S37. (**a) Photograph of **BN-Dira** nanoparticles dissolved in ddH_2_O. (b) TEM image of **BN-Dira** **NPs** dissolved in ddH_2_O (scale bar = 200 nm). (c) Particle size distribution diagram of **BN-Dira NPs**. (d) Photograph of **BN-Tetra** nanoparticles dissolved in ddH_2_O. (e) TEM image of **BN-Tetra NPs** dissolved in ddH_2_O (scale bar = 200 nm). (f) Particle size distribution diagram of **BN-Tetra NPs**. (g) UV-vis absorption spectra of **BN-Dira** and **BN-Dira NPs**. (h) UV-vis absorption spectra of **BN-Tetra** and **BN-Tetra NPs**.

**S8. Photothermal performance**

All animal experiments were performed according to the protocol approved by the Animal Care and Use Committee (CC/ACUCC) of Xiamen University (No. XMULAC20250048)

**Mice and cell lines**

All animal procedures were approved by the Institutional Animal Care and Use Committee of Xiamen University (approval No. XMULAC20250048). Female BALB/c mice, aged 7 to 8 weeks, were obtained from Vital River Laboratory Animal Technology Co., Ltd. (Beijing, China). The 4T1 murine breast carcinoma cell line and B16 murine melanoma cell line were provided by the National Institute of Diagnostics and Vaccine Development in Infectious Diseases (Xiamen University). 4T1 cells were used for both in vitro cytotoxicity assays and subcutaneous tumor model establishment, whereas B16 cells were used exclusively for in vitro experiments. Both cell lines were cultured in Dulbecco’s modified Eagle’s medium (DMEM, Gibco) supplemented with 10% fetal bovine serum (FBS, Gibco), 100 U mL⁻¹ penicillin, and 100 μg mL⁻¹ streptomycin (Invitrogen). All cell lines were routinely tested for mycoplasma contamination and confirmed to be negative before use.

**Statistical analysis**

Animal experiments were performed with randomized group allocation. Statistical comparisons were conducted using unpaired two‑tailed Student’s t test for two-group comparisons, or one‑way and two‑way analysis of variance (ANOVA) as appropriate, followed by Bonferroni’s post hoc test for multiple comparisons. For photothermal therapy studies, tumor volumes and body weights were analyzed using two‑way ANOVA with repeated measures where applicable. All data were assessed for normality using the Shapiro–Wilk test, and homogeneity of variances was verified using Levene’s test. GraphPad Prism (version 8.0.1) was used for all statistical analyses. A *p* value of < 0.05 was considered statistically significant, with significance levels indicated as ns (*p* ≥ 0.05), ****p* < 0.001, and *****p* < 0.0001. Data are presented as mean ± SD, with the number of biologically independent samples (n) specified in each figure legend. No outliers were excluded from the reported experiments.

**Calculation of photothermal conversion efficiency.** The photothermal conversion efficiency (η) was calculated according to Equation (1), according to published studies from Roper and Hu *et. al.*^[3,4]^

$\eta= \frac{hS \left( T_{max}- T_{surr} \right)- Q_{diss}}{I (1-{10}^{-A\lambda})}$ (1)

Where ℎ is the heat transfer coefficient, S is the irradiated area, *T_max_* is the maximum temperature of the sample, and *T_surr_* is the temperature of the surroundings. Q_diss_ is heat dissipation from the system to the surroundings, which was calculated to be 0.06 W for **BN-Dira** and 0.13 W for **BN-Tetra**, I is the laser incident power, A_λ_ is the absorbance of samples at a wavelength of 808 nm. Here, hS can be obtained by Equation (2)

$hS = \frac{m\cdot C_{p}}{\tau_{s}}$ (2)

In which m and Cp represent the total mass and heat capacity of the system, respectively. Given that the nanomaterials are dispersed in ddH₂O at low concentrations, their contribution to the overall heat capacity is negligible. Therefore, m corresponds to the mass of the solvent (ddH₂O), and Cp is taken as the specific heat capacity of water (4.2 J g⁻¹ °C⁻¹). The τ_s_ is the system time constant, calculated by Equation (3)

$t= -\tau_{s}ln\theta= -\tau_{s} ln \left( \frac{T-T_{surr}}{T_{max}- T_{surr}} \right)$ (3)

t is the time of the cooling process after irradiation.

In this work, τ_s_ was calculated as 136.98 s for **BN-Dira** and 174.81 s for **BN-Tetra**, as obtained from the linear fitting in Figure S38:

**Figure S38.** Calculation of photothermal conversion efficiency of a) **BN-Dira NPs** and b) **BN-Tetra NPs**.

For **BN-Dira**, the values of m and *C_p_* are 0.5 g and 4.2 J/(g·°C), respectively, and *A_808nm_*, I, and (*T_max_ -T_surr_*) are measured as 1.296, 0.924 W, and 51.78 °C, respectively. For **BN-Tetra**, the values of m and *C_p_* are 0.5 g and 4.2 J/(g·°C), respectively, and *A_808nm_*, *I*, and (*T_max_ -T_surr_*) are measured as 0.495, 0.304 W, and 22.74 °C, respectively. Above all, the photothermal conversion efficiency of **BN-Dira** and **BN-Tetra** was calculated to be 83.6 % and 69.3%, respectively.

**Figure S39.** A) Temperature-time profiles of **BN-Tetra NPs** (0.50 mg/mL) under 808 nm laser irradiation with varying power. B) Temperature-time profiles of **BN-Tetra NPs** at different concentrations under 808 nm laser (1.0 W/cm²) irradiation. C) Photothermal stability monitoring of **BN-Tetra NPs** (0.25 mg/mL) under 808 nm laser irradiation (0.8 W/cm²). D) Temperature-time profile of **BN-Tetra NPs** aqueous solution (0.25 mg/mL) after 5 min of 808 nm laser (0.8 W/cm²) irradiation and subsequent cessation. E) Calculation of photothermal conversion efficiency of **BN-Tetra NPs**. F) Infrared thermal imaging of **BN-Tetra NPs** at various concentrations under 808 nm laser irradiation (1.0 W/cm²). G) Infrared thermal imaging of **BN-Tetra NPs** (0.50 mg/mL) under 808 nm laser irradiation at different power levels.

**Figure S40**. A) Cytotoxicity of **BN-Dira NPs** and B) **BN-Tetra NPs** toward B16 cells under 808 nm laser irradiation (1.0 W/cm^2^), and C) DAPI/PI staining images of B16 cells subjected to different treatments. The data are shown as the mean ± SD from a representative experiment of two to three independent experiments with n = 4 biologically independent samples per group (a, b). Statistical significance was calculated via unpaired Student's t test (two tailed) comparing laser off versus laser on at the same material concentration, with **** indicating *p* < 0.0001.

**Figure S41.** HE-stained histological images of the heart, liver, spleen, lungs, and kidneys from mice in different treatment groups.

**Table S6.** PCE of small organic molecular PTAs in the reported literatures.

**S9. References**

1. Jing Jiang, Dandan Yuan, Congzhe Ma, Wanbin Song, Yaoyu Lin, Lihong Hu, and Yinan Zhang. *Org. Lett.* **2021** 23 (2), 279-284.

2. Frisch M, Trucks G, Schlegel H, Scuseria G, Robb M, Cheeseman J, *et al.* Gaussian 16, Revision C. 01. Gaussian, Inc., Wallingford CT. 2016.

3. D. K. Roper, W. Ahn, M. Hoepfner, *J. Phys. Chem. C* **2007**, 111, 3636-3641.

4. Q. Tian, F. Jiang, R. Zou, Q. Liu, Z. Chen, M. Zhu, S. Yang, J. Wang, J. Wang, J. Hu, *ACS Nano* **2011**, 5, 9761-9771.

5. S. Lee, S. Min, G. Kim, S. Lee, *Coord. Chem. Rev.* **2024**, 506, 215719.

6. Z. Li, H. Wen, X. Xiang, S. Sheng, T. Zhang, K. Ye, Z. Xie, C. Dou, *Sci. China Chem.* **2026***,* 69, 339–346.

7. Y-H. Zhong, G-F. Huang, S-Y. Zhao, L-H. Chung, H-T. Zhang, J-H. Zheng, Y-L. Yan, W-X. Ni, J. He, *Adv.Sci.* **2024**, 11, 2309068.

8. H. Gao, Y. Yao, C. Li, J. Zhang, H. Yu, X. Yang, J. Shen, Q. Liu, R. Xu, X. Gao, D. Ding, *Angew. Chem. Int. Ed.* **2024**, 63, e202400372.
